# Association Between Depression, Anxiety, and Antidepressant Use With T-Wave Amplitude and QT-Interval

**DOI:** 10.3389/fnins.2018.00375

**Published:** 2018-06-05

**Authors:** Mandy X. Hu, Femke Lamers, Brenda W. J. H. Penninx, Eco J. C. de Geus

**Affiliations:** ^1^Department of Psychiatry, Amsterdam Public Health Research Institute, VU University Medical Center, Amsterdam, Netherlands; ^2^Department of Biological Psychology, VU University, Amsterdam, Netherlands

**Keywords:** autonomic nervous system, cardiac repolarization, depression, anxiety, antidepressant

## Abstract

**Objectives:** Cardiac repolarization may be affected by psychiatric disorders and/or antidepressant use, but evidence for this is inconclusive. This study examined the relationship between depressive and anxiety disorder and use of antidepressants with T-wave amplitude (TWA) and QT-interval.

**Methods:** Data was obtained from the Netherlands Study of Depression and Anxiety (*n* = 1,383). Depression/anxiety was diagnosed with the DSM-IV based Composite International Diagnostic Interview. The use of tricyclic antidepressants (TCAs), selective serotonin and noradrenalin reuptake inhibitors (SNRIs), and selective serotonin reuptake inhibitors (SSRIs) was established. T-wave amplitude and QT-interval corrected for heart rate (QTc) were obtained from an ECG measured in a type II axis configuration.

**Results:** Compared to controls, persons with depression or anxiety disorders did not show a significantly different TWA (*p* = 0.58; Cohen's *d* = 0.046) or QTc (*p* = 0.48; Cohen's *d* = −0.057). In spite of known sympathomimetic effects, TCA use (*p* = 0.26; Cohen's *d* = −0.162) and SNRI use (*p* = 0.70; Cohen's *d* = −0.055) were not significantly associated with a lower TWA. TCA use (*p* = 0.12; Cohen's *d* = 0.225) and SNRI use (*p* = 0.11; Cohen's *d* = 0.227) were also not significantly associated with a prolonged QTc.

**Conclusion:** We did not find evidence that either depressive/anxiety disorder or antidepressant use is associated with abnormalities in TWA or QTc. Earlier found sympathomimetic effects of TCAs and SNRIs are not evident in these measures of cardiac repolarization.

## Introduction

Depressive and anxiety disorders have been associated with an increased risk of cardiovascular disease (CVD) (Nicholson et al., 2006; Vogelzangs et al., 2010), cardiovascular mortality and sudden death (Frasure-Smith and Lespérance, 2008). Dysregulation of the autonomic nervous system (ANS) may be part of the underlying mechanism linking these psychiatric disorders to unfavorable health outcomes (Holsen et al., 2012; Penninx et al., 2013). However, in view of the increasing evidence that the use of antidepressants negatively impacts autonomic balance (Davidson et al., 2005; Udupa et al., 2011), it has been suggested that the found effects of psychopathology on ANS activity are driven by medication use (O'Regan et al., 2015).

Studies linking cardiac autonomic dysregulation to mental health and antidepressant use have mostly used heart rate variability (HRV) as a measure of parasympathetic activity (Agelink et al., 2002; Bär et al., 2004; Shinba, 2014; Keen et al., 2015; Noordam et al., 2015; O'Regan et al., 2015; Borrione et al., 2017), and (nor)epinephrine concentrations (Zimmermann-Viehoff et al., 2014; Paine et al., 2015) or pre-ejection period (Silvia et al., 2016; Ahles et al., 2017; Diamond and Fisher, 2017) as a measure of sympathetic activity. Less focus is directed to measures of repolarization, such as T-wave amplitude (TWA) and QT-interval. TWA is the asymmetrical wave in the electrocardiogram following the QRS complex, and reflects repolarization of the ventricles (Haarmark et al., 2010). Sympathetic stimulation has been suggested to lead to a decreased TWA, whereas the role of the parasympathetic nervous system remains unclear (van Lien et al., 2015). QT-interval is defined as the interval from the onset of the QRS complex (the earliest indication of ventricular depolarization) to the end of the T wave (the latest indication of ventricular repolarization; Lepeschkin and Surawicz, 1952). Because the QT-interval is directly influenced by HR, it is recommended to use a measure corrected for HR: QTc (Brouwer et al., 2003). Increased sympathetic activity has been found to prolong QTc (Magnano et al., 2002; Baumert et al., 2008). Both abnormalities in TWA (Tan et al., 2008; Okuda et al., 2011) and prolonged QTc (Okin et al., 2000; Mozos and Serban, 2011) have been associated with cardiac morbidity and mortality. If these measures are affected by psychiatric disorders and/or antidepressant use, they could explain part of the comorbidity between poor mental health and cardiovascular disease. Previous studies concerning QT variables and psychiatric disorders have tentatively confirmed this relationship. One study investigated QTc in relation to depression-related personality, and found a positive relationship (Minoretti et al., 2006). Another study concluded that depressive symptoms were non-significantly associated with prolonged QTc (Kamphuis et al., 2007). Studies by the group of Bär and Koschke (Bär et al., 2008; Koschke et al., 2009) have investigated patients with schizophrenia and major depressive disorder and found an increase in QT variability (dynamic changes in QT-interval duration) in these patients. However, they did not report on measures of QT-interval. Both QT variability and QT-interval have been associated with increased risk of cardiac mortality (Berger et al., 1997; Okin et al., 2000). However, a study by Baumert et al. (2008) did not find a correlation between these measures, suggesting that they reflect different parts of the repolarization dynamics. Interestingly, the same study found a positive correlation between norepinephrine spillover and QTc, but not QT variability, in patients with depression or panic disorder, providing more evidence that these measures are influenced by differential mechanisms. To our knowledge, no study has linked basal QTc or TWA to depressive or anxiety disorder. Considering antidepressant use, studies have indicated that the use of tricyclic antidepressants (TCA) are associated with a lower TWA and a prolonged QTc (Burckhardt et al., 1978; Burgess et al., 1979; Giardina et al., 1979; Wilens et al., 1996; Zemrak and Kenna, 2008), while the effects of selective serotonergic and noradrenergic reuptake inhibitors (SNRIs) and selective serotonin reuptake inhibitors (SSRIs) are less conclusive (Roos, 1983; Glassman et al., 2002; Raskin et al., 2003; Zhang et al., 2007; Baumert et al., 2008; Isbister, 2009; Castro et al., 2013).

Whether psychopathology is independently associated with autonomic dysregulation or whether this association is driven by antidepressant use, remains an ongoing debate (Agelink et al., 2001; Kemp et al., 2012; O'Regan et al., 2015). This study aimed to investigate the association between depression/anxiety and antidepressant use with TWA and QTc.

## Methods

### Subjects

Data was obtained from NESDA, a longitudinal cohort study including 2,981 subjects recruited from community, primary care, and mental health care in the Netherlands. The participants were aged 18–65 and consisted of people with a current diagnosis of depression and/or anxiety disorder, a history of these disorders, and healthy controls. Baseline measurement was conducted between 2004 and 2007, and follow-up assessments took place after 2, 4, 6, and 9 years. A detailed description of the rationale, objectives and methods of the NESDA study can be found elsewhere (Penninx et al., 2008). The study protocol was performed conform the declaration of Helsinki and approved by the Ethical Review Board of each participating center. All participants provided written informed consent.

Data were drawn from the 9-year follow-up of the NESDA study (*n* = 2,069), conducted between 2014 and 2017. Of the total sample, 686 participants were excluded because they had missing physiological data (due to telephone or at-home interviews without ANS recording, equipment failure during assessment, or poor electrocardiogram quality). This resulted in a total of 1,383 participants for analyses. Excluded participants were older and were more often treated with cardiac medication.

### Depressive/anxiety disorder

Participants were diagnosed with the DSM-IV based Composite International Diagnostic Interview (CIDI), version 2.1 (Wittchen, 1994), and divided into three groups: (1) a control group with no lifetime history of psychiatric disorders, (2) a remitted psychopathology group with major depressive disorder (MDD) or anxiety disorder (panic disorder, social phobia, and/or generalized anxiety disorder) earlier in life but not in the past 6 months, (3) a current psychopathology group with either MDD, anxiety disorder, or both in the past 6 months.

In addition to diagnosis, the severity of depression and anxiety was measured in all participants using the 30-item Inventory of Depressive Symptomatology, Self-Report (IDS-SR) (Rush et al., 1996) and the Beck Anxiety Inventory (BAI) (Beck et al., 1988).

### Antidepressant use

Medication use was determined by inspection of medication containers that participants brought to the assessment. Participants were considered to be currently using antidepressants when they reported to have used medication frequently (daily or more than 50% of the time) in the past month. We divided the participants into four groups: (1) non-users, (2) persons using TCAs (ATC code N06AA), (3) persons using SSRIs (ATC code N06AB), and (4) persons using SNRIs (ATC code N06AX). There were four persons using TCAs and SSRIs simultaneously, and 1 person using both TCA and SNRI. Since TCAs have been found to have the strongest effect on cardiac autonomic activity (Licht et al., 2010, 2012), we decided to group these persons under TCA use. There was one person using SNRI and SSRI at the same time, whom we grouped under SNRI use, since SNRIs have been found to have a stronger effect on cardiac autonomic activity than SSRIs (Licht et al., 2010, 2012). A derived daily dose for antidepressant use was calculated by dividing the participant's mean daily dose by the daily dose recommended by the World Health Organization (World Health Organization, 2008).

### Physiological measurements

Physiological data were recorded with an unobtrusive lightweight portable device containing a six-electrode configuration: the “Vrije Universiteit Ambulatory Monitoring System” (VU-AMS). This device measures electrocardiograms (ECG) and impedance cardiography (ICG) (De Geus and Van Doornen, 1996). Heart rate (HR) was derived from the ECG interbeat interval (IBI) time series (Neijts et al., 2014). TWA was calculated by subtracting the amplitude of T-offset from the point of highest amplitude of T-wave deflection (van Lien et al., 2015). QT-interval was defined as the interval from the onset of the QRS complex (the earliest indication of ventricular depolarization) to the end of the T wave (the latest indication of ventricular repolarization; Lepeschkin and Surawicz, 1952). Since the QT-interval is directly influenced by HR, a corrected measure was calculated using Bazett's formula (QTc = QT/√RR) (Brouwer et al., 2003).

VU-AMS software (version 3.8, VU University Amsterdam, www.vu-ams.nl) was used for data cleaning. Non-stationary periods were detected by movement registration through vertical accelerometry and removed. Bad ECG signal fragments (artifacts) were automatically detected, after which a modified version of the algorithm by Christov (2004) was used to detect R-wave peaks. Participants were excluded when their recordings showed more than 5% highly suspicious IBIs of the total recording. An event marker was used to divide the assessment into four different conditions: a supine rest condition with blood pressure measurement (±11 min) and three sitting conditions: a psychiatric interview (±45 min), a general interview (±47 min), and a computer task (±7 min). The ECG signals were ensemble averaged across these conditions by time locking them to the R-wave peaks (van Lien et al., 2015). Exploratory mixed model analyses showed that there were no significant interaction effects between psychopathology status and assessment condition for TWA (*p* = 0.98) or QTc (*p* = 0.84), nor between antidepressant groups and condition for TWA (*p* > 0.99) or QTc (*p* > 0.99). Therefore, data during the four conditions were collapsed to create a single average score of HR, TWA, QT-interval, and QTc, with an average total recording duration of ±107 min.

### Covariates

Adjustments were made for sociodemographics: age, sex, and years of education. We also accounted for health factors: physical activity measured by the International Physical Activity Questionnaire (Booth et al., 2003), alcohol use (units per week), number of smoked cigarettes/day, body mass index (BMI), number of treated chronic diseases (cardiovascular disease, epilepsy, diabetes, osteoarthritis, stroke, cancer, chronic lung disease, thyroid disease, liver disease, intestinal disorders, and ulcer) and use of cardiac medication (ATC codes C01, C02, C03, C04, C05, C07, and C08).

### Statistical analyses

Data were analyzed using SPSS, version 22.0. Characteristics across the psychopathology groups were compared using ANOVA analyses, Kruskal–Wallis one-way analysis of variance, and χ2-statistics. Analyses of covariance (ANCOVA) were used to investigate the relationship between psychopathology diagnosis and antidepressant use with cardiac autonomic variables. Cohen's *d* effect sizes were calculated, defined as the difference in the means of two groups, divided by the pooled standard deviation of these groups. Regression analyses were used to establish the association between cardiac autonomic activity and IDS-SR score, BAI score, and derived daily dose of antidepressant use. ANCOVA and regression analyses were adjusted for age, sex, and education in a first model, and for physical activity, alcohol use, smoking, BMI, number of chronic diseases, and cardiac medication in a second model. Analyses with psychopathology were additionally corrected for antidepressant use in a third model. The criterion for statistical significance was *p* < 0.05.

## Results

Our sample (*n* = 1,383) had a mean age of 51.0 years (*SD* = 13.1) and 64.4% were female. Of the participants, 26.6% had a current depressive/anxiety disorder, 52.4% had a history of depression/anxiety, and 19.7% used antidepressants (Table 1).

**Table 1:** Sample characteristics.

**Sociodemographic**	**Total sample (*n =* 1383)**	**Controls (*n =* 290)**	**Remitted depression/anxiety (*n =* 725)**	**Current depression/anxiety (*n =* 368)**	***p***
Age, years (mean ±*SD*)	51.0 ± 13.1	50.3 ± 14.6	51.6 ± 13.1	50.4 ± 11.7	0.21
Female (%)	64.4	58.6	65.1	67.7	0.048
Education, years (mean ±*SD*)	13.1 ± 3.3	13.8 ± 3.3	13.0 ± 3.2	12.9 ± 3.4	0.001
**LIFESTYLE AND HEALTH**					
Physical activity, 1000METmin/week [median (IQR)]	2.9 (1.5–5.4)	2.9 (1.7–5.6)	3.1 (1.6–5.7)	2.6 (1.3–4.8)	0.005
Alcohol use, drinks/week [median (IQR)]	3.7 (0.2–8.2)	3.7 (0.4–8.2)	3.7 (0.2–8.2)	1.0 (0.2–8.2)	0.001
Smokers (%)	23.6	15.2	25.8	26.1	0.001
No. cigarettes/day [median (IQR)]	10.0 (4.0–15.0)	8.3 (3.0–14.8)	10.0 (4.0–15.0)	10.0 (4.3–20.0)	0.20
BMI (mean ±*SD*)	26.3 ± 4.8	25.9 ± 4.8	26.3 ± 4.6	26.8 ± 5.3	0.074
No. chronic diseases					<0.001
0 (%)	52.9	64.8	50.9	47.6	
1–2 (%)	42.1	32.1	44.6	44.9	
>2 (%)	5.1	3.1	4.5	7.7	
Use of cardiac medication (%)	16.9	17.6	17.0	16.3	
**MENTAL HEALTH**					
Psychopathology					
Current (%)	26.6	–	–	–	
Remitted (%)	52.4	–	–	–	
IDS-SR score [median (IQR)]	12.0 (6.0–21.0)	5.0 (2.0–10.0)	12.0 (7.0–18.0)	24.0 (15.5–33.5)	<0.001
BAI-score [median (IQR)]	5.0 (2.0–11.0)	1.0 (0.0–4.0)	5.0 (2.0–9.0)	12.0 (6.0–20.0)	<0.001
Use of TCA (%)	3.7	0.0	3.2	7.6	<0.001
TCA DDD (mean ± SD)	1.0 ± 0.8	–	0.9 ± 0.8	1.1 ± 0.7	0.42
Use of SNRI (%)	3.8	0.0	4.4	5.7	<0.001
SNRI DDD (mean ±*SD*)	1.1 ± 0.7	–	1.0 ± 0.7	1.3 ± 0.7	0.16
Use of SSRI (%)	12.2	0.0	11.9	22.6	<0.001
SSRI DDD (mean ±*SD*)	1.2 ± 0.6	–	1.1 ± 0.5	1.3 ± 0.7	0.012

*IQR, interquartile range; METs/min, multiple of resting metabolic rate times minutes of physical activity per week; BMI, body mass index; IDS-SR, Inventory of Depressive Symptomatology-Self Report; BAI, Beck Anxiety Inventory; TCA, tricyclic antidepressant; SNRI, selective serotonergic and noradrenergic reuptake inhibitors; SSRI, selective serotonin reuptake inhibitors; TWA, T-wave amplitude; HR, heart rate; QTc, heart rate corrected QT-interval*.

*Comparison using ANOVA analyses (normally distributed continuous variables), Kruskal-Wallis one-way analysis of variance (non-normally distributed continuous variables), and χ2-statistics (categorical variables)*.

Table 2 shows the association between depressive/anxiety disorder and ANS values. No significant association was seen between depression/anxiety and TWA. Compared to controls, people with remitted (*p* = 0.033; Cohen's *d* = −0.150) and current depression/anxiety (*p* = 0.002; Cohen's *d* = −0.254) had a significantly lower HR in the fully adjusted model. In addition, people with current depression/anxiety had a significantly longer QT-interval (fully adjusted model: *p* = 0.018; Cohen's *d* = 0.192). However, this association disappeared after correcting QT-interval for HR (QTc). When analyzing the association between IDS-SR and BAI score with ANS values (Table 3), no significant results were found after full adjustment.

**Table 2:** TWA and QTc values in patients with remitted or current depressive/anxiety disorder compared to controls.

	**Controls *n* = 290**		**Remitted depression/anxiety *n* = 725**				**Current depression/anxiety *n* = 368**			
**ANS value**	**Mean**	**SE**	**Mean**	**SE**	***p*^a^**	**Cohen's *d*^a^**	**Mean**	**SE**	***p*^a^**	**Cohen's *d*^a^**
TWA (mV)^b^	1.219	0.036	1.220	0.023	0.99	0.002	1.219	0.032	>0.99	−0.001
TWA (mV)^c^	1.214	0.036	1.213	0.022	>0.99	−0.002	1.236	0.031	0.64	0.036
TWA (mV)^d^	1.211	0.036	1.213	0.022	0.97	0.003	1.239	0.032	0.58	0.046
QT-interval (ms)^b^	380.297	1.741	380.513	1.095	0.92	0.007	383.803	1.539	0.13	0.119
QT-interval (ms)^c^	379.490	1.734	380.378	1.081	0.67	0.030	**384.704**	**1.531**	**0.026**	**0.177**
QT-interval (ms)^d^	379.318	1.760	380.267	1.074	0.65	0.032	**385.058**	**1.550**	**0.018**	**0.192**
HR (beats/min)^b^	71.424	0.563	70.679	0.354	0.26	−0.078	70.508	0.498	0.22	−0.096
HR (beats/min)^c^	71.937	0.554	70.719	0.346	0.063	−0.130	**70.025**	**0.489**	**0.011**	−**0.203**
HR (beats/min)^d^	72.159	0.556	**70.763**	**0.339**	**0.033**	−**0.150**	**69.763**	**0.490**	**0.002**	−**0.254**
QTc^b^	412.986	1.546	410.944	0.973	0.27	−0.078	413.113	1.367	0.95	0.005
QTc^c^	413.539	1.545	410.908	0.964	0.15	−0.101	412.749	1.364	0.70	−0.030
QTc^d^	413.950	1.580	410.912	0.964	0.10	−0.115	412.417	1.392	0.48	−0.057

*TWA, T-wave amplitude; QTc, heart rate corrected QT-interval*.

a *p-values and cohen's d were compared to controls*.

b *Analyses of covariance adjusted for age, sex, and education*.

c *Adjustment^b^ + additionally adjusted for physical activity, alcohol use, smoking, BMI, number of chronic diseases, and use of cardiac medication*.

d *Adjustment^c^ + additionally adjusted for antidepressant use*.

*Boldface indicates statistical significance (p < 0.05)*.

**Table 3:** Associations between IDS-SR and BAI scores with TWA and QTc values.

	**IDS-SR score *n* = 1342**		**BAI score *n* = 1341**	
**ANS value**	**β**	***p***	**β**	***p***
TWA (mV)^a^	−0.005	0.85	−0.028	0.28
TWA (mV)^b^	0.017	0.58	−0.015	0.60
TWA (mV)^c^	0.022	0.49	−0.010	0.73
QT-interval (ms)^a^	0.014	0.62	−0.024	0.38
QT-interval (ms)^b^	−0.006	0.86	−**0.062**	**0.049**
QT-interval (ms)^c^	0.009	0.79	−0.043	0.17
HR (beats/min)^a^	0.030	0.28	**0.068**	**0.013**
HR (beats/min)^b^	0.040	0.22	**0.087**	**0.005**
HR (beats/min)^c^	0.015	0.65	0.055	0.074
QTc^a^	0.034	0.20	0.034	0.20
QTc^b^	0.030	0.35	0.017	0.57
QTc^c^	0.023	0.48	0.009	0.76

*IDS-SR, Inventory of Depressive Symptomatology-Self Report; BAI, Beck Anxiety Inventory; TWA, T-wave amplitude; HR, heart rate; QTc, heart rate corrected QT-interval*.

a *Regression analyses were adjusted for age, sex, and education*.

b *Adjustment^a^ + additionally adjusted for physical activity, alcohol use, smoking, BMI, number of chronic diseases, and use of cardiac medication*.

c *Adjustment^b^ + additionally adjusted for antidepressant use*.

*Boldface indicates statistical significance (p < 0.05)*.

Table 4 shows the association between antidepressant use and ANS values. Although users of TCAs had a lower TWA, this difference was not statistically significant (fully adjusted model: *p* = 0.26; Cohen's *d* = −0.162). Compared to non-users, TCA users had a significantly higher HR (fully adjusted model: *p* = < 0.001; Cohen's *d* = 1.048) and shorter QT-interval (fully adjusted model: *p* = < 0.001; Cohen's *d* = −0.661). However, after correction for HR, TCA use (fully adjusted model: *p* = 0.12; Cohen's *d* = 0.225) and SNRI use (fully adjusted model: *p* = 0.11; Cohen's *d* = 0.227) were non-significantly associated with a longer QTc-interval. When investigating the relationship between antidepressant derived daily dose (DDD) and ANS values (Table 5), we found an unexpected lower TWA in subjects with higher DDD of SSRI (fully adjusted model: β = −0.177; *p* = 0.022).

**Table 4:** TWA and QTc values in persons using antidepressants compared to non-users.

	**Non-users** ***n*** = **1,116**		**TCA-users** ***n*** = **51**				**SNRI-users *n* = 53**				**SSRI-users *n* = 169**			
**ANS value**	**Mean**	**SE**	**Mean**	**SE**	***p*^a^**	**Cohen's *d*^a^**	**Mean**	**SE**	***p*^a^**	**Cohen's *d*^a^**	**Mean**	**SE**	***p*^a^**	**Cohen's d^a^**
TWA (mV)^b^	1.229	0.018	1.092	0.086	0.12	−0.225	1.162	0.086	0.45	−0.110	1.212	0.048	0.74	−0.028
TWA (mV)^c^	1.224	0.018	1.126	0.085	0.26	−0.162	1.191	0.084	0.70	−0.055	1.224	0.048	>0.99	−0.001
QT-interval (ms)^b^	381.490	0.876	**362.294**	**4.120**	**<0.001**	−**0.654**	383.001	4.096	0.72	0.052	385.722	2.281	0.084	0.145
QT-interval (ms)^c^	381.620	0.873	**362.240**	**4.123**	**<0.001**	−**0.661**	383.193	4.070	0.71	0.054	384.802	2.313	0.20	0.108
HR (beats/min)^b^	70.461	0.280	**80.518**	**1.315**	**<0.001**	**1.073**	72.263	1.307	0.18	0.193	69.546	0.728	0.24	−0.098
HR (beats/min)^c^	70.478	0.276	**80.181**	**1.303**	**<0.001**	**1.048**	72.179	1.286	0.20	0.185	69.564	0.731	0.25	−0.098
QTc^b^	411.278	0.783	418.002	3.682	0.075	0.256	417.234	3.661	0.11	0.228	412.988	2.039	0.43	0.065
QTc^c^	411.410	0.783	417.328	3.701	0.12	0.225	417.335	3.653	0.11	0.227	412.266	2.076	0.70	0.032

*TWA, T-wave amplitude; HR, heart rate; QTc, heart rate corrected QT-interval; TCA, tricyclic antidepressant; SNRI, selective serotonergic and noradrenergic reuptake inhibitors; SSRI, selective serotonin reuptake inhibitors*.

a *p-values and cohen's d were compared to non-users*.

b *Analyses of covariance adjusted for age, sex, and education*.

c *Adjustment^b^ + additionally adjusted for physical activity, alcohol use, smoking, BMI, number of chronic diseases, use of cardiac medication, and psychopathology*.

*Boldface indicates statistical significance (p < 0.05)*.

**Table 5:** Associations between antidepressant derived daily dose (DDD) and TWA and QTc values.

	**TCA *n* = 51**		**SNRI *n* = 52**		**SSRI *n* = 167**	
**ANS value**	**β**	***p***	**β**	***p***	**β**	***p***
TWA (mV)^a^	0.126	0.37	−0.017	0.90	−**0.224**	**0.003**
TWA (mV)^b^	0.091	0.51	0.020	0.91	−**0.177**	**0.022**
QT-interval (ms)^a^	−0.050	0.73	−0.060	0.67	−0.044	0.57
QT-interval (ms)^b^	0.041	0.79	−0.222	0.20	0.006	0.94
HR (beats/min)^a^	0.154	0.30	0.11	0.45	0.106	0.18
HR (beats/min)^b^	0.001	>0.99	0.35	0.053	0.052	0.53
QTc^a^	0.088	0.54	0.007	0.96	0.070	0.36
QTc^b^	0.071	0.65	0.022	0.90	0.086	0.28

*TWA, T-wave amplitude; HR, heart rate; QTc, heart rate corrected QT-interval; TCA, tricyclic antidepressant; SNRI, selective serotonergic and noradrenergic reuptake inhibitors; SSRI, selective serotonin reuptake inhibitors*.

a *Regression analyses adjusted for age, sex, and education*.

b *Adjustment^a^ + additionally adjusted for physical activity, alcohol use, smoking, BMI, number of chronic diseases, use of cardiac medication, and psychopathology*.

*Boldface indicates statistical significance (p < 0.05)*.

To test whether the results were not influenced by differences in signal processing, we compared ECG artifacts and respiration rate across the groups. When comparing current psychopathology (0.29%), remitted psychopathology (0.33%), and healthy controls (0.30%), we found no difference in percentage of ECG artifacts (*p* = 0.59). A difference was found when comparing ECG artifacts between users of TCA (0.60%), SNRI (0.25%), SSRI (0.23%), and non-users (0.32%) (*p* = 0.003). This difference was caused by a higher percentage of artifacts in TCA users. However, even in this group the percentage was still very low, making it unlikely that artifacts influenced the results substantially. When comparing respiration rate across the different psychopathology and antidepressant groups, we did not find a significant difference. In addition, when we corrected for respiration rate in the analyses, the results remained similar.

## Discussion

The current study did not find an association between depressive/anxiety disorder and TWA and QT-interval, two measures of cardiac repolarization that are affected by sympathetic activity and associated with cardiac morbidity and mortality (Mozos and Serban, 2011; Okuda et al., 2011). In spite of their known adrenergic effects, TCA and SNRI use were not associated with a lower TWA or a prolonged QTc.

Regarding psychopathology, we did find an association between a current depressive/anxiety disorder and a prolonged QT-interval. However, this result was driven by a lower HR in these patients, since the association rendered non-significant when using the HR-adjusted QTc. Importantly, when investigating severity scores of depression and anxiety, we did not find any associations with the TWA or QTc measures after adjustment for antidepressants. These null-findings are somewhat surprising as previous studies have shown an association between T wave (Whang et al., 2014) and QT (Minoretti et al., 2006; Koschke et al., 2009) abnormalities with depressive and anxious symptomatology. However, two of these studies investigated different aspects of these variables (T wave inversion or QT variability), and the third investigated the relationship of QTc with depression-related personality. To the best of our knowledge, no study has investigated the relationship between TWA and QTc with diagnosed depression and anxiety. Our findings are in line with research by Kamphuis et al. (2007), indicating that there were no significant associations between QTc and depressive symptoms, and with previous NESDA research claiming that psychopathology might not have a negative impact on sympathetic activity as assessed using pre-ejection period, independent from antidepressant use (Licht et al., 2012).

The current study could not confirm findings suggesting that the use of TCAs is associated with a lower TWA and a prolonged QTc (Burgess et al., 1979; Giardina et al., 1979; Wilens et al., 1996; Zemrak and Kenna, 2008). Most of the previous studies investigated the acute effects of short-term medication use on cardiac repolarization. In our study, we examined patients who were generally using TCAs for a longer term. Perhaps the effect of short-term TCA use on QTc and TWA diminishes with chronic use. This theory is in line with a study by Burckhardt et al. (1978), indicating a modest effect of TCA use on TWA and QTc after 3 weeks, which disappeared after 13 months of antidepressant use. Considering SNRI use, our results indicate that there is no effect of this group of antidepressants on cardiac repolarization, congruent with several studies that found none to minor changes in QTc in patients using duloxetine or venlafaxine (Raskin et al., 2003; Zhang et al., 2007; Isbister, 2009). These null-findings of TCA and SNRI use are in contrast to previous NESDA studies suggesting strong associations between the use of these antidepressants with higher HR and shorter pre-ejection period, indicating increased sympathetic activity (Licht et al., 2012; Hu M. et al., 2017). However, it is important to bear in mind that different mechanisms of cardiac autonomic activity are in play concerning the investigated variables. Whereas, HR and pre-ejection period are measures of sympathetic effects on sinoatrial node pacemaker activity and contractility of the ventricles respectively, TWA and QTc are measures of sympathetic effects on repolarization of the ventricles. That these measures do not reflect the exact same mechanism is illustrated by a study by van Lien et al. (2015), showing a correlation of only 0.4 between TWA and pre-ejection period. We suggest that there are sympathomimetic effects of TCAs and SNRIs which are apparent in HR and pre-ejection period, but not in TWA and QTc.

Studies on the relationship between SSRI use and measures of cardiac repolarization have generally concluded that this group of antidepressants has no significant effect on QTc or TWA (Roos, 1983; Glassman et al., 2002; Baumert et al., 2008). Our study indicated that SSRI users did not differ from controls in repolarization measures, but we found a lower TWA in SSRI users that had higher SSRI DDD. An effect of SSRI on repolarization would be consistent with a study by Castro et al. (2013), that found a dose-response relationship between several SSRIs (citalopram and escitalopram) with a prolonged QTc. However, since there was no general effect of SSRI use on TWA, the found association between a higher SSRI DDD and lower TWA might be a chance finding.

### Limitations of the study

In order to interpret the results of the current study, some limitations need to be taken into account. We recorded ANS measures during several conditions, but not during a true rest condition, rendering this measure prone to all sources of variability. However, exploratory mixed model analyses showed that there were no significant interaction effects between psychopathology status/antidepressant group and assessment condition for TWA or QTc. Also, when comparing respiration rate between the psychopathology groups and antidepressant groups vs. controls, we found no significant differences for any of the conditions. In addition, we have shown in a previous study that the average of the conditions has good temporal stability for measures of HR, respiratory sinus arrhythmia, and pre-ejection period (Hu M. et al., 2017), suggesting that we are assessing robust and reliable variables.

Unfortunately, in our study we did not measure other aspects of cardiac repolarization, such as QT variability, or T-wave inversions. Previous studies have associated psychopathology with increased QT variability (Yeragani et al., 2000; Bär et al., 2008; Koschke et al., 2009). A study by Whang et al. (2014) showed that depressive and anxious symptoms were associated with abnormalities in T wave inversions. Similarly, there are many more mechanisms of repolarization to be considered, such as spatial and temporal dispersion of repolarization, configuration of the action potential, and early post-depolarization. For instance, prolongation of the action potential or variations in duration can produce lability in the repolarization process, which may manifest as variability or dispersion of repolarization duration, or early after depolarizations that can initiate triggered arrhythmias (Tomaselli et al., 1994). Investigating these different aspects would further the understanding of the underlying mechanisms linking mental health to cardiovascular risk.

Several studies have implied altered ANS reactivity to stressors among depressed patients rather than ANS dysregulation during rest conditions (Sheffield et al., 1998; Hughes and Stoney, 2000). The current study did not contain a stressor or intervention to investigate the association between psychopathology or antidepressant use on reactivity of QTc or TWA. A next step would be to investigate such reactivity measures (e.g., active standing or a stress task) for QTc and TWA in relation to depression and anxiety.

Medication use and its derived daily dose were determined by inspection of medication containers that participants were asked to bring to the assessment session and self-report. Since psychiatric patients have shown low adherence to treatment (DiMatteo et al., 2000), we cannot establish for certain that the reported (amount of) medication was taken by the participants, which might have influenced the results. In addition, the NESDA study only contains outpatient participants, who likely use lower dosage of antidepressants than inpatient persons. The current study did not include many participants who used antidepressants in a high dosage (DDD was equal or <1.5 for 99.3% persons who used TCA, 99.5% persons who used SNRI, and 97.4% persons who used SSRI). Therefore, we cannot rule out an effect of antidepressant overuse on cardiac repolarization. Indeed, studies have shown that especially TCA overdose was associated with ventricular tachycardia and abnormalities in TWA and QTc (Langou et al., 1980; Niemann et al., 1986; Thanacoody and Thomas, 2005). However, here we aimed to investigate the effects of therapeutic use of antidepressants on cardiac repolarization, and did not find evidence for a significant relationship. Another limitation is that we only had data of TWA and QTc at one wave of data collection, which confined us to a cross-sectional design where we could only compare antidepressant users to non-users. Longitudinal data would have allowed investigation of within-subject effects of antidepressant use (e.g., the effects of chronically using, starting, or stopping the use of medication) and might have rendered more conclusive results.

These limitations are balanced by several strengths. This is one of few studies to investigate the relationship between depressive and anxiety disorders with TWA and QTc. This study was conducted within a large sample of participants recruited from community, primary care, and mental health care. Compared to healthy controls, persons with poor mental health often have poorer lifestyle (e.g., less physically active, more often smokers, higher BMI) and physical health (e.g., more chronic diseases and use of medication; Molarius et al., 2009; Scott and Happell, 2011). Previous research has shown that these factors are also drivers of autonomic change over time (Hu M. et al., 2017; Hu M. X. et al., 2017). Our large sample size allowed the correction for these important confounders.

## Conclusions

In summary, in our large cohort study we did not find evidence that either depressive/anxiety disorder or antidepressant use is associated with abnormalities in TWA or QTc. Earlier findings of the sympathomimetic effects of TCAs and SNRIs on HR and pre-ejection period are not evident in these measures of cardiac repolarization.

## Ethics statement

This study was carried out in accordance with the recommendations for the use of human subjects, VUmc Medical Ethical Review Board. The protocol was approved by the VUmc Medical Ethical Review Board. All subjects gave written informed consent in accordance with the Declaration of Helsinki.

## Author contributions

MH formulated the research question, performed statistical analyses, wrote the manuscript, and incorporated feedback from all co-authors. FL provided feedback in all drafts of the manuscript and critically interpreted the results. BP and EdG reviewed and provided feedback for the research question, provided feedback in all drafts of the manuscript, and critically interpreted the results.

### Conflict of interest statement

The authors declare that the research was conducted in the absence of any commercial or financial relationships that could be construed as a potential conflict of interest.

## Abbreviations

ANS: autonomic nervous system
BAI: Beck Anxiety Inventory
BMI: body mass index
CIDI: Composite International Diagnostic Interview
CVD: cardiovascular disease
DDD: derived daily dose
ECG: electrocardiogram
HR: heart rate
HRV: heart rate variability
IBI: interbeat interval
ICG: impedance cardiography
IDS-SR, Inventory of Depressive Symptomatology: Self-Report
IQR: interquartile range
METmin: multiple of resting metabolic rate times minutes of physical activity per week
MDD: Major depressive disorder
NESDA: Netherlands Study of Depression and Anxiety
QTc: HR corrected QT-interval
SNRI: selective serotonin and noradrenalin reuptake inhibitors
SSRI: selective serotonin reuptake inhibitors
TCA: tricyclic antidepressant
TWA: T-wave amplitude
VU-AMS: Vrije Universiteit Ambulatory Monitoring System.
